# Nicotinamide-Expanded Allogeneic Natural Killer Cells with CD38 Deletion, Expressing an Enhanced CD38 Chimeric Antigen Receptor, Target Multiple Myeloma Cells

**DOI:** 10.3390/ijms242417231

**Published:** 2023-12-07

**Authors:** Avishay Edri, Nimrod Ben-Haim, Astar Hailu, Nurit Brycman, Orit Berhani-Zipori, Julia Rifman, Sherri Cohen, Dima Yackoubov, Michael Rosenberg, Ronit Simantov, Hideshima Teru, Keiji Kurata, Kenneth Carl Anderson, Ayal Hendel, Aviad Pato, Yona Geffen

**Affiliations:** 1 Gamida-Cell, Jerusalem 34670, Israel; avishaye@gamida-cell.com (A.E.); astarlazmi@gmail.com (A.H.); nurit101272@gmail.com (N.B.); oberhani@gmail.com (O.B.-Z.); ykbio3@gmail.com (J.R.); scohen929@gmail.com (S.C.); dimay@gamida-cell.com (D.Y.); aviadpa@gmail.com (A.P.); 2 Institute of Nanotechnology and Advanced Materials, The Mina and Everard Goodman Faculty of Life Sciences, Bar-Ilan University, Ramat-Gan 52900, Israel; nimrod5ben@gmail.com (N.B.-H.); michael.rosenberg@biu.ac.il (M.R.); 3 Gamida-Cell, Boston, MA 02116, USA; ronit@gamida-cell.com; 4 Jerome Lipper Multiple Myeloma Center, LeBow Institute for Myeloma Therapeutics, Dana-Farber Cancer Institute, Harvard Medical School, Boston, MA 02215, USA; teru_hideshima@dfci.harvard.edu (H.T.); keiji_kurata@dfci.harvard.edu (K.K.); kenneth_anderson@dfci.harvard.edu (K.C.A.)

**Keywords:** natural killer cell, cell therapy, immunotherapy, chimeric antigen receptor, multiple myeloma, CD38, NAM platform, fratricide

## Abstract

Natural killer (NK) cells are a vital component of cancer immune surveillance. They provide a rapid and potent immune response, including direct cytotoxicity and mobilization of the immune system, without the need for antigen processing and presentation. NK cells may also be better tolerated than T cell therapy approaches and are susceptible to various gene manipulations. Therefore, NK cells have become the focus of extensive translational research. Gamida Cell’s nicotinamide (NAM) platform for cultured NK cells provides an opportunity to enhance the therapeutic potential of NK cells. CD38 is an ectoenzyme ubiquitously expressed on the surface of various hematologic cells, including multiple myeloma (MM). It has been selected as a lead target for numerous monoclonal therapeutic antibodies against MM. Monoclonal antibodies target CD38, resulting in the lysis of MM plasma cells through various antibody-mediated mechanisms such as antibody-dependent cellular cytotoxicity (ADCC), complement-dependent cytotoxicity, and antibody-dependent cellular phagocytosis, significantly improving the outcomes of patients with relapsed or refractory MM. However, this therapeutic strategy has inherent limitations, such as the anti-CD38-induced depletion of CD38-expressing NK cells, thus hindering ADCC. We have developed genetically engineered NK cells tailored to treat MM, in which CD38 was knocked-out using CRISPR-Cas9 technology and an enhanced chimeric antigen receptor (CAR) targeting CD38 was introduced using mRNA electroporation. This combined genetic approach allows for an improved cytotoxic activity directed against CD38-expressing MM cells without self-inflicted NK-cell-mediated fratricide. Preliminary results show near-complete abolition of fratricide with a 24-fold reduction in self-lysis from 19% in mock-transfected and untreated NK cells to 0.8% of self-lysis in CD38 knock-out CAR NK cells. Furthermore, we have observed significant enhancements in CD38-mediated activity in vitro, resulting in increased lysis of MM target cell lines. CD38 knock-out CAR NK cells also demonstrated significantly higher levels of NK activation markers in co-cultures with both untreated and αCD38-treated MM cell lines. These NAM-cultured NK cells with the combined genetic approach of CD38 knockout and addition of CD38 CAR represent a promising immunotherapeutic tool to target MM.

## 1. Introduction

Multiple myeloma (MM) is an aggressive B cell malignancy. It accounts for 1% of all cancers and approximately 10% of all hematologic malignancies. Each year, over 32,000 new cases are diagnosed in the United States, and almost 13,000 patients die of the disease [1,2]. The treatment of MM is mainly based on alkylators and corticosteroids, immunomodulatory drugs, proteasome inhibitors, monoclonal antibodies (mAbs), and histone deacetylase inhibitors. Nevertheless, relapsed and refractory MM still poses a major challenge [3,4]. To address this need, new therapeutic strategies are being developed in the field of cellular immunotherapy [5]. Currently, there are only two cell therapies approved by the US FDA for the treatment of MM. Both are autologous T-cell-based therapies, genetically modified to express a chimeric antigen receptor (CAR), directed against the B cell maturation antigen (BCMA) [6]. Ongoing clinical trials are employing NK cells for the treatment of MM. Tested NK-cell-based immunotherapies are mostly in an allogeneic, off-the-shelf setting. Allogeneic NK cell immunotherapies have been shown to augment the lymphocyte population, which, in turn, contributes to the prolongation of progression-free survival (PFS), and are well tolerated with no evidence of graft versus host disease (GvHD) [7].

NK cells are a key component of cancer immune surveillance [8,9,10,11]. They provide a rapid and potent immune response, including direct cytotoxicity and mobilization of the immune system, without the need for antigen processing and presentation. Moreover, they are easy to isolate and expand, considered relatively safe, and are not associated with a risk of cytokine storm or GvHD [12,13,14]. Therefore, NK cells have become the focus of extensive translational research aimed at leveraging their antitumor activity along with their safety profile and susceptibility to various gene manipulations to create a safe and effective immunotherapeutic tool for the treatment of hematologic malignancies and solid tumors [9,15,16,17].

CD38 is ubiquitously expressed on the surface of plasma cells and is also found on NK cells, B cells, and T cells [18,19]. It has been selected as a target for numerous monoclonal therapeutic antibodies targeting MM cells, such as Elotuzumab and Daratumumab, as targeting CD38 has been shown to facilitate the lysis of MM plasma cells through various antibody-mediated mechanisms such as antibody-dependent cell cytotoxicity (ADCC), complement-dependent cytotoxicity (CDC), and antibody-dependent cellular phagocytosis (ADCP), significantly improving the outcomes of patients with relapsed or refractory MM [20,21,22,23,24]. However, this therapeutic strategy has some inherent limitations, such as anti-CD38-induced depletion of CD38-expressing NK cells, which in turn hinders ADCC [25,26,27].

Here, we report a novel immunotherapeutic strategy based on the benefits of the previously established nicotinamide (NAM)-cultured NK platform. These are cryopreserved, genetically engineered NK cells tailored for the treatment of MM, in which CD38 has been knocked-out using CRISPR-Cas9 technology, and an enhanced CAR-targeting CD38 has been introduced using mRNA electroporation. This allows an improved cytotoxic activity directed against CD38-expressing MM cells without any self-inflicted lysis by NK cells (fratricide).

## 2. Results

### 2.1. CRISPR-Based CD38 Knock-Out in Primary NK Cells Is Precise and Efficient

**Figure 1:**
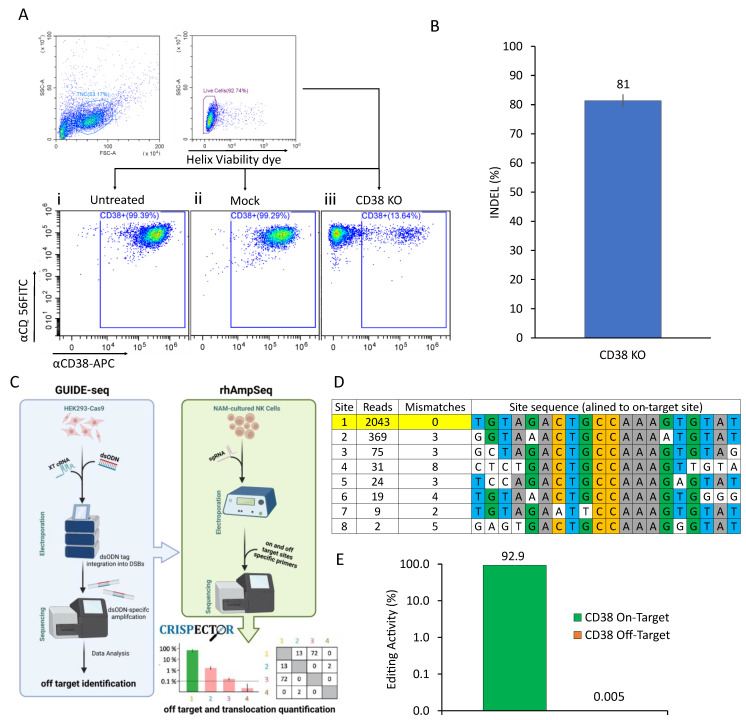
**CD38 Knock-out Editing Strategy.** (**A**) CD38 knock-out phenotyping. On the day of harvest, untreated (i), mock (ii) and CD38 KO (iii) cells were stained with FITC-conjugated anti-CD56 mAb and an APC-conjugated anti-CD38 mAb followed by the addition of the Helix viability dye immediately prior to analysis by flow cytometry. (**B**) CD38 knock-out was performed using a CRISPR-Cas9-based system. Human primary NK cells were electroporated with 4uM RNP-complex, targeting CD38. To evaluate editing efficiency, DNA from the cells was extracted after 14 days in culture. Sanger sequencing was performed and analyzed via TIDE to determine INDEL frequencies. CD38 sgRNA yields a high editing rate (81% INDEL frequency), mean ± SD, n = 3. (**C**) An illustration describing the NGS experiments performed for the nomination (GUIDE-Seq) and quantification (rhAmpSeq) of off-target activity followed by CRISPECTOR software analysis (version 1.0.5). (**D**) GUIDE-Seq analysis. Double-stranded oligodeoxynucleotides (dsODNs) were co-delivered together with CD38 XT two-part gRNA to a Cas9-expressing HEK293 stable cell line. The intended target sequence is shown in the top line with cleaved sites shown underneath and with mismatches to the on-target site shown and highlighted in white. GUIDE-seq sequencing read counts and the number of mismatches are shown to the left of each site. (**E**) CD38 on- and off-target editing levels were determined by rhAmpSeq in CD38 knock-out CAR NK cells. Cas9 was complexed with CD38 sgRNA at a 1:2.5 Cas9:gRNA molar ratio. rhAmpSeq data were analyzed using CRISPECTOR software (version 1.0.5). The editing threshold is 0.1%. Editing percentages designated above the bars. The on-target bar is colored in green. Error bars represent a 95% confidence interval. The results shown are from one representative experiment of five performed.

While there is evidence of activity for NAM-cultured alloreactive NK cells in patients with non-Hodgkin lymphoma [28,29,30], the treatment of MM may require adjustments to be made to compensate for its suppressive nature and lack of natively expressed stimulatory molecules [31,32,33,34,35,36]. Some myeloma-specific antigens have been previously identified and targeted in various therapeutic strategies, including mAbs and CAR-bearing immune effector cells [24,25,37,38,39,40,41]. The first-in-class mAb to target MM was Daratumumab, an anti-CD38 mAb which was shown to facilitate lysis of MM plasma cells through various antibody-mediated mechanisms such as ADCC, CDC, and ADCP, significantly improving the outcomes of patients with relapsed or refractory myeloma. However, high expression of CD38 in both untreated NK cells (Figure 1(Ai)) and mock-electroporated NK cells (Figure 1(Ai)) may limit the potential benefit of using NK cells in combination with a CD38-targeting mAbs such as Daratumumab due to the possibility of anti-CD38-induced depletion of CD38-expressing NK cells, known as fratricide. This effect, in turn, may hinder ADCC against MM cells. Aiming to eliminate this destructive mechanism, we have used CRISPR-Cas9 gene editing technology to knock-out CD38 expression in our primary NK cells. We have designed a set of seven gRNA sequences targeting different areas of the CD38 gene (Figure S1A). Cells were electroporated with a CRISPR-Cas9 RNP complex targeting CD38, and the editing efficiency was evaluated by Sanger sequencing and analyzed using Tracking Indels by Decomposition (TIDE) to determine insertions and deletions (INDEL) frequencies (Figure 1B) [42]. On our selected gRNA 5, we observed a high editing rate of up to 81% INDEL frequency (Figure S1B). This was also manifested in the phenotypic analysis performed by flow cytometry, showing a gradual reduction in CD38 throughout the two-week culture, peaking at approximately 90% knock-out on day 14 (Figure 1(Aiii)). As a complementary step following the above efficiency evaluation, we analyzed the safety of our CRISPR-Cas9 gene editing procedure by assessing the likelihood of off-target editing effects. To do so, we utilized a rigorous two-step evaluation procedure (Figure 1C). First, we employed the GUIDE-seq method to establish a permissive nomination of possible off-target cleavage sites (OTSs) across the genome [43]. This initial analysis revealed only seven possible OTSs (Figure 1D). Subsequently, we used rhAmpSeq to quantify off-target activity in edited cells, focusing on the previously detected OTSs [44]. We analyzed the resulting data using CRISPECTOR software (version 1.0.5) [45] and observed a high editing activity of 92.9% at the intended on-target site, with no detectable off-target activity (Figure 1E) or translocations.

### 2.2. CD38 Knock-Out Eliminates αCD38-Mediated Fratricide

**Figure 2:**
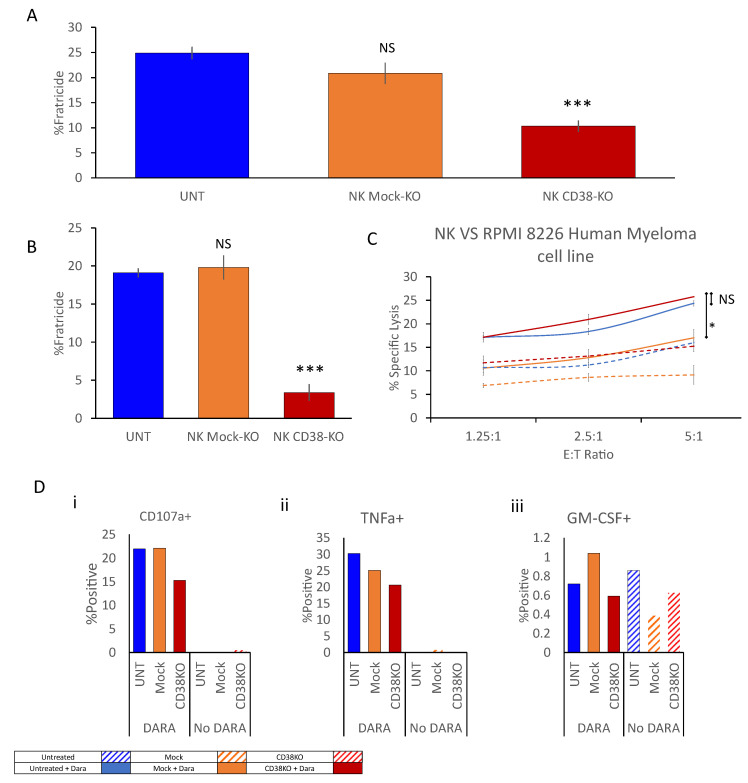
**CD38 knock-out eliminates anti-CD38-mediated fratricide.** Untreated, mock-electroporated, and CD38 knock-out NK cells were simultaneously thawed and co-cultured with CFSE-labelled RPMI 8226 cells either in the presence or absence of Daratumumab at E:T ratios varying from 5:1 to 0:1 for 6 h. Cells were harvested and stained for viability by Helix viability dye. (**A**,**B**) CFSE-negative NK cells were gated, and the percentage of dead NK cells was determined; spontaneous NK cell death was subtracted to calculate fratricide levels. (**A**) In the absence of target cells. (**B**) Within an NK vs. RPMI8266 co-culture. (**C**) Alternatively, target cells were CFSE-gated, and the percentage of dead cells was determined, and spontaneous target cell death was subtracted to calculate specific target cell lysis levels. (**D**) Thawed NK cells were co-cultured with RPMI 8226 cells either in the presence or absence of Daratumumab at an E:T ratio of 1:3 for 6 h. Cells were then stained for CD107a (i), TNFα (ii), and GM-CSF (iii) by flow cytometry. Dead cells were excluded by Zombie violet viability dye; NK cells were gated by staining for CD56 and the relative percentage of activation-marker-expressing cells was determined. Results shown are from one representative experiment of three performed NS = Non-significant * *p* < 0.05, *** *p* < 0.0005 compared with UNT + Daratumumab. Student’s t test, comparison to the nearest bar.

To evaluate the functional significance of CD38 knock-out, we designed an array of fluorescence-activated cell sorting (FACS)-based functional assays to measure the fratricide level within cultured NK cells in the presence of Daratumumab at a concentration of 1 ug/mL. Fratricide was measured both in co-culture with target cells and an NK culture with no target cells present. Fratricide levels in untreated and mock-electroporated cells increased up to 25%; total fratricide was reduced by 2.5-fold to 10% within the CD38 knock-out NK culture (Figure 2A). In the presence of RPMI-8226 target cells, fratricide levels in the untreated and mock-electroporated cell culture were reduced to approximately 20% compared to the level measured without target cells. The fratricide level measured in the CD38 knock-out culture co-cultured with RPMI-8226 target cells was reduced by approximately 5.5-fold to 3.3% (Figure 2B). This significant reduction in fratricide did not result in a corresponding increase in the specific lysis of target cells by NK cells (Figure 2C) nor did it cause an increase in CD107a expression (Figure 2(Di)) or an increase in measured tumor necrosis factor (TNF)α or granulocyte-macrophage colony-stimulating factor (GM-CSF) (Figure 2(Dii,iii)).

### 2.3. Anti-CD38 CAR Expression Results in Extensive Fratricide and Effector Cell Exhaustion

**Figure 3:**
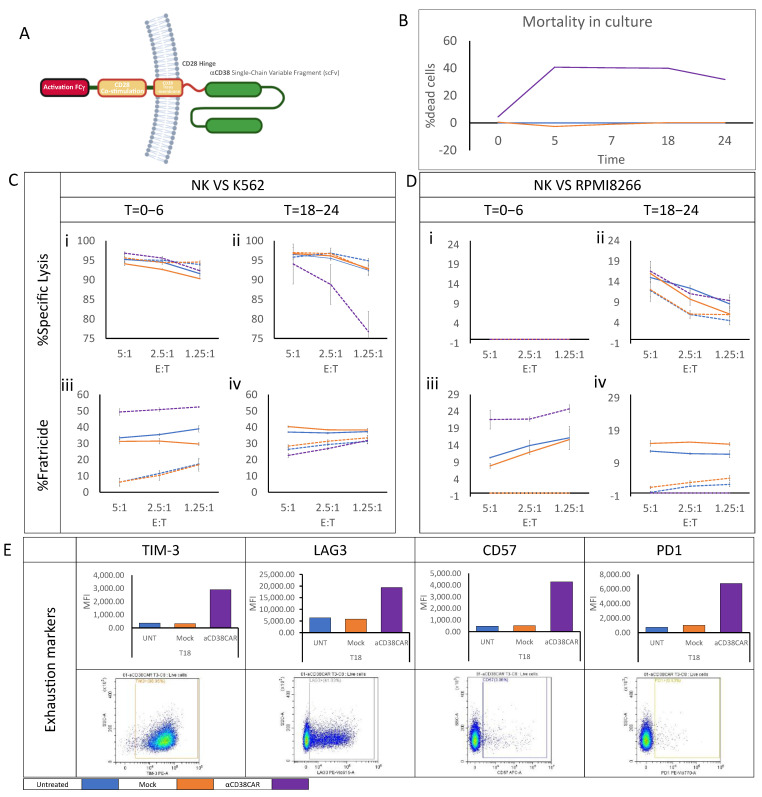
**Anti-CD38 CAR expression results in extensive fratricide and effector cell exhaustion.** Peripheral blood mononuclear cells were obtained by apheresis from a healthy volunteer and purified by CD3-negative selection using the CliniMACS system. The CD3-negative fraction was cultured for 14 days in an NAM-supplemented medium in the presence of an irradiated CD3-positive fraction as feeder cells and IL-15. On the day of harvest, an mRNA anti-CD38 CAR was introduced into cells by electroporation. (**A**) Illustration of anti-CD38 CAR. Alternatively, cells were either mock-electroporated or left untreated. Immediately after electroporation, cells were seeded in separate flasks containing complete growth media supplemented with IL2, cells were sampled on designated timepoints, and viability was recorded. (**B**) Mortality of untreated NK cells was determined as background spontaneous cell mortality. (**C,D**) Untreated, mock-electroporated and CAR-electroporated cells were sampled at two timepoints (T = 0 h (i,iii), T = 18 h (ii,iv) post electroporation) and co-cultured with CFSE-labelled RPMI 8226 or K562 cells either in the presence or absence of Daratumumab at E:T ratios varying from 5:1 to 0:1 for 6 h. Cells were harvested and stained for viability by Helix viability dye. CFSE-negative NK cells were gated, and the percentage of dead NK cells was determined, spontaneous NK cell death was subtracted to calculate fratricide levels. Alternatively, target cells were CFSE-gated, the percentage of dead cells was determined, and spontaneous target cell death was subtracted to calculate specific target cell lysis levels. (**E**) Cells sampled on the final timepoint (T = 24) were stained with R-phycoerythrin-conjugated anti-TIM3, PE-Vio 615-conjugated anti-LAG3, Allophycocyanin-conjugated anti-CD57 and PE-Vio 770-conjugated anti-PD1 to determine the expression of selected exhaustion markers. The bottom plot displays the percentage of positive cells in CAR-expressing cells; the top plot displays the mean fluorescence intensity (MFI) of positive cells in each tested NK cell group. The results shown are from one representative experiment out of three performed.

Considering these findings, we sought to increase the direct responsiveness of NK cells to MM target cells. To do so, we designed an NK CAR customized with an FCγ activation motif, which is naturally occurring in NK cells. In addition, we included a CD28-based co-stimulatory motif and hinge, all fused to an mAb-based anti-CD38 Single-Chain Variable Fragment (Figure 3A). To evaluate the effect of anti-CD38 CAR expression on NK cells, a two-step functional assay was used in which NK cells were cultured for two weeks under standard conditions in the presence of NAM and harvested. Cells were then either electroporated with anti-CD38 CAR mRNA, mock-electroporated, or left untreated; cell groups were plated in three different flasks and incubated in a 37 °C incubator for 24 h (Figure S2). During this incubation, cells were sampled five times and stained for viability. As expected, mortality levels were distinctly higher in the anti-CD38 CAR-expressing culture due to fratricide. To isolate mortality specifically caused by fratricide, we determined lysis levels in untreated cells as spontaneous background lysis levels. The fratricide level measured in the anti-CD38 CAR-expressing culture rapidly increased during the first 6 h of incubation and remained steady at 40% mortality for the following 18 h. In the measurement taken 24 h post-electroporation, a 10% decrease in fratricide levels was observed, possibly resulting from the complete disintegration of some dead cells in the culture (Figure 3B). In parallel, NK cells from each flask were sampled immediately after electroporation and 18 h post-electroporation. Cells were subsequently co-cultured with K562 (CD38-) and RPMI8266 (CD38+) for six hours in the presence or absence of Daratumumab. Interestingly, in NK cells co-cultured with K562 target cells, the specific lysis levels measured six hours post-electroporation were comparable in all groups at approximately 90% regardless of Daratumumab pretreatment (Figure 3(Ci)). The lysis assay starting 18 h post-electroporation showed a reduction in CAR-expressing NK cell activity to 77% lysis of target cells (Figure 3(Cii)). Fratricide levels measured six hours post-electroporation, however, were remarkably high in CAR-expressing NK cells, reaching as high as 50%. In untreated and mock-electroporated NK cells co-cultured in the presence of Daratumumab, fratricide levels increased by approximately 35%. Predictably, changes in fratricide levels in untreated and mock-electroporated NK cells without Daratumumab were minimal (Figure 3(Ciii)). While fratricide levels measured 24 h post-electroporation remained stable in untreated and mock-electroporated NK cells, fratricide levels in CAR-expressing NK cells were reduced to basal levels similar to the level measured in untreated and mock-electroporated NK cells without Daratumumab (Figure 3(Civ)). When co-cultured with RPMI8266 cells, target cell lysis in all NK co-cultures was initially undetectable (Figure 3(Di)). The lysis assay starting 18 h post-electroporation showed some specific target cell lysis with a minor increase in lysis by the untreated and mock-electroporated NK groups in the presence of Daratumumab and by anti-CD38 CAR-expressing NK cells compared to other groups tested (Figure 3(Dii)). Nevertheless, substantial fratricide levels were detected in both co-culture assays conducted after electroporation and 18 h post-electroporation. Fratricide in untreated and mock-electroporated NK groups co-cultured in the presence of Daratumumab ranged from 10 to 15%, while fratricide levels remained negligible in the absence of Daratumumab. Interestingly, fratricide levels measured in the anti-CD38 CAR-expressing NK cell co-culture in the absence of Daratumumab were notably high on the assay performed immediately after electroporation, increasing up to 25% (Figure 3(Diii)), and were reduced in the assay performed 18 h post-electroporation to the basal level measured in the untreated and mock-electroporated NK cell groups in the absence of Daratumumab (Figure 3(Div)). These observations correspond with changes detected in the expression of several exhaustion markers, such as TIM-3 and LAG-3, which were clearly elevated in the anti-CD38 CAR-expressing NK cells compared to untreated and mock-electroporated cells 18 h post-electroporation. CD57 and PD1 expressing cells, although very few, also had increased expressions (Figure 3E).

### 2.4. CD38 Knock-Out NK Cells Expressing Anti-CD38 CAR Show Enhanced Anti-Tumor Activity against MM Cell Lines

**Figure 4:**
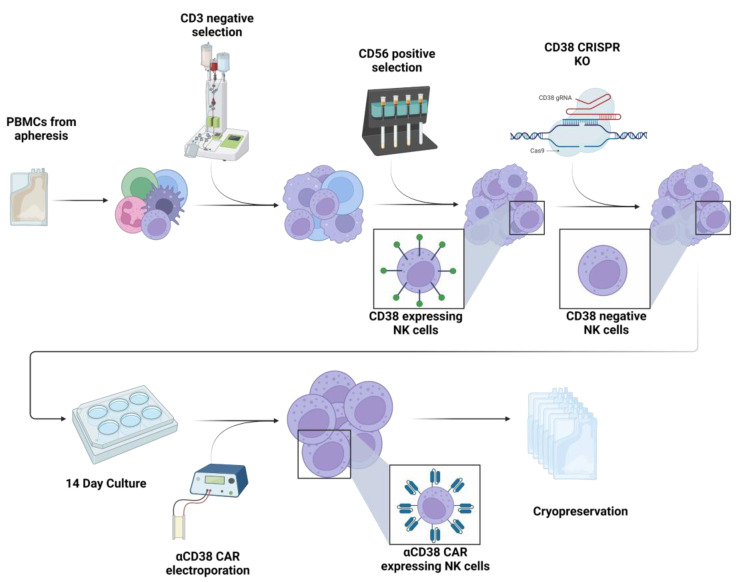
**CD38 knock-out CAR production process.** Peripheral blood mononuclear cells were obtained by apheresis from a healthy volunteer. Cells were purified by CD3-negative selection using a CliniMACS system followed by a CD56-positive selection using MACS cell separation columns. After a short recuperation in IL2-supplemented media, the purified cells were subjected to CRISPR-Cas9 editing aimed to knock-out CD38. CD38 knock-out cells were then cultured for 14 days in an NAM-supplemented media in the presence of irradiated feeder cells and IL-15. On the day of harvest, an mRNA anti-CD38 CAR was introduced into cells by electroporation. Approximately 6 h post electroporation, the cells were cryopreserved.

Based on accumulated data emphasizing the significance of fratricide in hindering NK cell function by leading to exhaustion or cell death, we devised a strategy to reduce fratricide and increase NK cell reactivity against CD38-expressing malignancies. As such, we proceeded to knock-out CD38 expression in NK cells followed by expression of the CD38 CAR (Figure 4).

**Figure 5:**
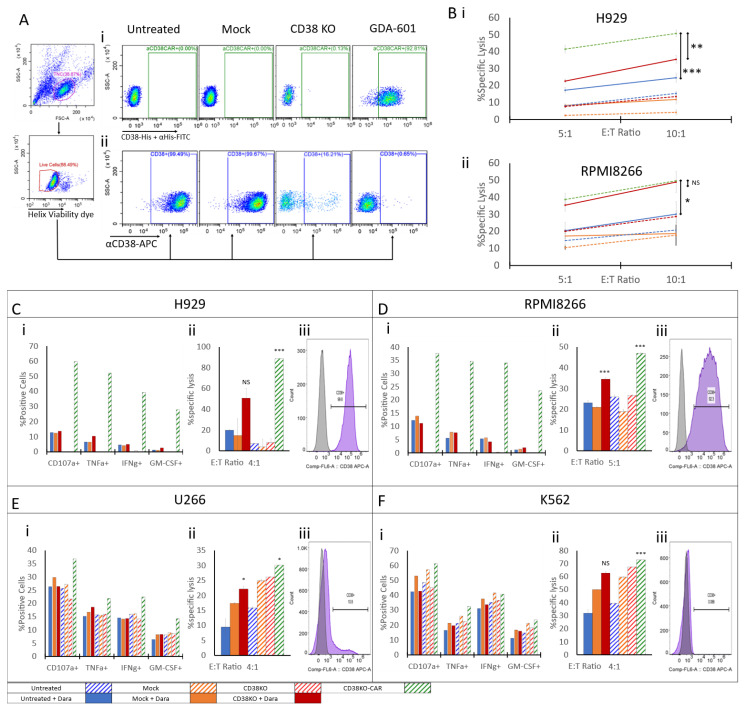
**CD38 knock-out CAR NK cells expressing anti-CD38 CAR show enhanced anti-tumor activity against MM cell lines.** (**A**) CD38 knock-out CAR NK cell phenotyping. On day of harvest, 6 h after electroporation, all effector cells were stained with FITC-conjugated anti-CD56 mAb and an APC-conjugated anti-CD38 mAb (i), alternatively, cells were incubated with His-tag-conjugated CD38 to induce complex formation with αCD38 CAR, followed by staining with FITC-conjugated αHis tag mAb (ii). Stainings were followed by addition of the Helix viability dye immediately prior to analysis by flow cytometry. (**B**) Thawed NK cells were co-cultured with calcein-AM-treated H929 (**Bi**) or RPMI 8226 (**Bii**) target cells either in the presence or absence of Daratumumab at E:T ratios of 5:1 and 10:1 for 4 h. The culture supernatants were collected and fluorescence was measured. (**Ci**,**Di**,**Fi**,**Ei**) Untreated, mock-electroporated and CD38 knock-out NK cells were simultaneously thawed and co-cultured with H929 (**Ci**), RPMI 8226 (**Di**), U266 (**Ei**) and K562 (**Fi**) target cells either in the presence or absence of Daratumumab at an E:T ratio of 1:3 for 6 h. Cells were then stained for CD107a, IFNγ, TNFα, and GM-CSF by flow cytometry. Dead cells were excluded by a Zombie violet viability dye; NK cells were gated by staining for CD56 and the relative % of activation-marker-expressing cells was determined. (**Cii**,**Dii**,**Fii**,**Eii**) Thawed NK cells were co-cultured with CFSE-labelled H929 (**Cii**), RPMI 8226 (**Dii**), U266 (**Eii**) and K562 (**Fii**) target cells either in the presence or absence of Daratumumab at E:T ratios of 4:1 for 6 h. Cells were harvested and stained for viability by Helix viability dye. Target cells were CFSE-gated, and the percentage of dead cells was determined; spontaneous target cell death was subtracted to calculate specific target cell lysis levels. (**Ciii**,**Diii**,**Fiii**,**Eiii**) Target cells were stained with APC-conjugated anti-CD38 mAb followed by Helix viability dye. The results shown are from one representative experiment of two performed. NS= Non-significant * *p* < 0.05, ** *p* < 0.005, *** *p* < 0.0005 compared with UNT + Daratumumab. Student’s *t* test.

Phenotyping was performed by flow cytometry in untreated, mock-electroporated, CD38 knock-out NK cells and CD38 knock-out CAR NK cells to measure the level of anti-CD38 CAR expression versus membrane-associated CD38 expression. To measure CAR expression, we used soluble biotin-tagged CD38 to bind the anti-CD38 CAR expressed on cells. PE-conjugated streptavidin was then used to fluorescently tag the anti-CD38 CAR-biotin CD38 complex. CAR expression levels on our CD38 knock-out CAR NK cells reached above 92% 6 h post electroporation (Figure 5(Ai)). CD38 expression levels measured in all conditions showed comparable results to those previously observed, with the knock-out level reaching as high as 90% reduction in CD38. Interestingly, CD38 expression on our CD38KO-CAR NK cells was eliminated completely and reduced to the background level (Figure 5(Aii)). Longitudinal measurements taken during the cell recovery stage revealed a gradual decrease in CD38 staining aligned with a consistent, gradual increase in anti-CD38 CAR expression (Figure S3). Functional assays were performed in all NK cell groups in the presence or absence of Daratumumab. A control group presenting anti-CD38 CAR-expressing NK cells without CD38 knock-out was excluded from the experimental design due to high toxicity, which manifested in vast fratricide as previously described [46,47] and as presented in Figure 5C, thus rendering it impossible to obtain the starting conditions required for a specific lysis assay. Testing included two independent methods of specific lysis assays and quantification of activation markers following co-culture with various target cells expressing different levels of CD38. First, NK cells were incubated in a co-culture with H929 myeloma cells, which naturally express high levels of CD38 (Figure 5(Ciii)). Activation levels measured in the absence of Daratumumab were minimal in all NK groups except for the CD38 knock-out CAR group. Nevertheless, some activation was detected once Daratumumab was introduced into the co-culture, reaching between 10% and 15% expression of the CD107a degranulation marker, 5% and 10% intracellular expression of TNFα, approx. 5% intracellular expression of interferon (IFN)γ, and 1% and 3% intracellular expression of GM-CSF. A slight increase in these markers was noted in CD38 knock-out NK cells. CD38 knock-out CAR NK cells, however, measured in the absence of Daratumumab demonstrated a significant response, with CD107a levels reaching as high as 60%, TNFα 52%, IFNγ 39%, and GM-CSF 28% (Figure 5(Ci)). Specific lysis of H929 target cells corresponded to the measured activation markers showing a very low level of specific lysis by untreated, mock electroporated, and CD38 knock-out NK cells in the absence of Daratumumab. As expected, some increase in specific lysis was observed in target cells co-cultured with NK cells in the presence of Daratumumab, increasing up to 20% in untreated and mock-electroporated cells and as high as 45% in CD38 knock-out NK cells. H929 lysis, induced by CD38 knock-out CAR NK cells, was remarkably high, peaking at 88% (Figure 5(Ci)). RPMI8266 target cells are generally characterized by a lower CD38 expression than that of H929 (Figure 5(Diii)). Accordingly, activation markers measured in all tested NK cell types without Daratumumab were negligible. In the presence of Daratumumab, NK cells co-cultured with RPMI8266 target cells showed some increase in activation similar to that observed in H929. However, CD38 knock-out CAR NK cells showed distinctively higher activation levels than all tested NK groups (Figure 5(Di)). The killing assay showed significantly reduced killing levels of RPMI8266 cells by all tested NK cell groups apart from CD38 knock-out CAR NK cells, which maintained their distinct capacity to engage and kill target cells. The U266 myeloma cell line is characterized by a low CD38 expression (Figure 5(Eiii)) along with some expression of NKG2D ligands [36]. Activation marker expression measured in NK cells co-cultured with U266 cells corresponded well with its reported phenotype. While some activation was noted in all NK cell groups, the addition of Daratumumab resulted in a minor increase in activation markers. CD38 knock-out CAR NK cells showed clear superiority over other NK cell groups even in the presence of Daratumumab (Figure 5(Ei)). The killing assay performed in a similar setting showed a slight increase in target cell lysis in CD38 knock-out CAR NK cell co-cultures compared to other NK groups incubated without Daratumumab. Interestingly, a reduction in target cell lysis was recorded in all NK cell groups when incubated in the presence of Daratumumab (Figure 5(Eii)). Lastly, functional assays were employed to assess the reactivity of CD38 knock-out CAR NK cells to CD38-negative K562 target cells (Figure 5(Fiii)). K562 cells are known to be human leukocyte antigen (HLA)-deficient cells expressing high levels of NKG2D and NKp30 ligands [48,49]; this phenotype makes K562 cells a prominent target for NK cells. When co-cultured with K562, NK cells of all tested groups showed elevated levels of activation markers, with a slight advantage in the absence of Daratumumab. With all tested activation markers, CD38 knock-out CAR NK cells showed a minor advantage over other tested NK groups despite the lack of CD38 expression in target cells (Figure 5(Fi)). Lysis assays maintained a similar trend, with notable lysis of K562 in all tested NK cell groups and a marginal increase in lysis in the absence of Daratumumab compared to the lysis measured in Daratumumab-treated target cells (Figure 5(Fii)). The cytotoxic activity of NK cells was also tested by a calcein-AM release assay, showing a significant increase in H929 target cell lysis by CD38 knock-out CAR cells compared to all other tested groups (Figure 5(Bi)), and an RPMI8266 lysis level slightly higher than that of CD38 knock-out cells in the presence of Daratumumab (Figure 5(Bii)).

### 2.5. aCD38 CAR Expression Allows Specific CD38-Targeted Activity While Maintaining Native NK-Expressed Receptor Function

**Figure 6:**
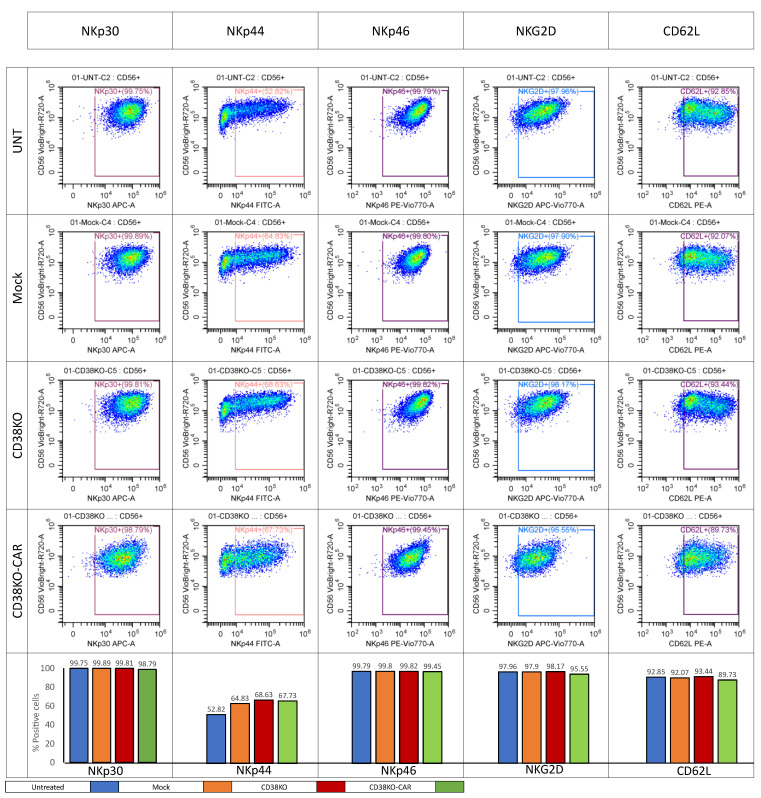
**Activating-receptor-expression phenotyping.** On the day of harvest, 6 h after electroporation, all effector cells were stained with a mix of fluorophore-conjugated mAbs directed to stain a panel of selected NK-expressed activating receptors, including APC-conjugated αNKp30 mAb, FITC-conjugated αNKp44 mAb, PE-Vio770-conjugated αNKp46 mAb, APC-Vio770-conjugated αNKG2D mAb, and PE-conjugated αCD62. Stainings were followed by the addition of the Helix viability dye immediately prior to analysis by flow cytometry.

We investigated the integrity of a select number of major activating interactions crucial for CAR-independent anti-tumoral activity by phenotypically analyzing NK cells using a panel of fluorophore-conjugated mAbs. An activating receptor panel and inhibiting receptor panel were designed and the cells were stained and subjected to flow cytometry. Tested activating receptors included NKp30, NKp44, NK46, NKG2D, and CD62L. All activating receptors exhibited stable expressions (Figure 6). Similarly, minimal variations were observed in the expression levels of tested inhibition markers. NKG2A and TIM3 showed high expressions across all four NK cell groups, while LAG3, PD-1, and TIGIT displayed low expression levels (Figure S5). To further validate the integrity of the natively expressed receptors, we designed an assay to evaluate NK activation mediated through a specific activation axis by immobilizing specific known ligands for NK-expressed receptors. Each antigen was serially diluted with an equal concentration of human serum albumin serving as a control protein solution to create a gradient in tested protein concentration, while the total protein concentration was kept steady (Figure S4). NK cells of various tested groups were cultured on the coated plates. Following culture, the expression of activation markers was measured. Initially, an anti-CD38 CAR was evaluated. A double gradient of CD38 coating was prepared; one gradient was pre-treated with Daratumumab, and the other was left untreated. NK cells cultured on CD38 gradients in the absence of Daratumumab showed no response to CD38. In the presence of Daratumumab, however, a notable response was recorded starting at a CD38/HSA ratio of 25:75 and very gradually intensifying in signal as the CD38 concentration increased. CD38 knock-out CAR NK cells showed a response superior to that measured on Daratumumab-pretreated CD38 gradients, with a linear, dose-dependent increase in activation signal throughout the gradient (Figure S4A). To test NKG2D and NKp30 gradients, MICA and B7H6 were used. These showed comparable levels of activation in all tested NK groups, including CD38 knock-out CAR cells (Figure S4B,C). Similarly, CD16 was tested by creating an HER2 gradient followed by Herceptin treatment to create an antibody–antigen complex. Similar activation was recorded in all tested NK groups, including CD38 knock-out CAR (Figure S4D).

## 3. Discussion

The emerging use of cell therapies for the treatment of MM in recent years has been a source of great promise [50]. While CAR-T-cell-based treatments have dominated the landscape, several innovative approaches have emerged. However, these approaches have encountered challenges, primarily characterized by a substantial rate of relapse. Some leading causes of this limitation are antigen escape, poor persistence of adaptively transferred cells, and the strong inhibitory nature of the tumor microenvironment. Additionally, the personalized manufacturing of autologous CAR T cells is highly expensive and time-consuming, often allowing disease progression and sometimes death while the patient is waiting to receive treatment [51,52]. Therefore, an off-the-shelf, potent, metabolically fit, and antigenically diverse cell therapy agent is in demand. Aiming to meet this demand, we developed a novel NAM-enhanced NK cell therapy agent with all validation experiments conducted in an off-the-shelf setting using cryopreserved cells.

We have chosen to direct our CAR to target CD38. CD38 is a prime target in MM, with two anti-CD38 mAbs (Daratumumab and Isatuximab) currently approved for use in the United States. These immunotherapeutic agents have transformed the treatment of MM, with a diverse mechanism of action composed of antibody-dependent, cell-mediated cytotoxicity, antibody-dependent cellular phagocytosis, and complement-dependent cytotoxicity [53,54,55,56]. While CD38 has exhibited susceptibility to immunoediting as a consequence of selective pressure imposed by immunotherapeutic agents [57], we have deliberately chosen to target it. Our rationale stems from the design of our CAR, which exhibits a heightened sensitivity, enabling it to identify myeloma cells expressing low levels of CD38, a phenomenon frequently observed in relapsed patients.

An inherent challenge associated with CD38-targeting therapies lies in the expression of CD38 on NK cells. This gives rise to fratricide, wherein NK cells undergo self-inflicted lysis due to their own CD38 expression. Initially, we tackled the issue of fratricide by genetically editing out the expression of CD38. We had anticipated that this modification by itself may be sufficient to significantly enhance the therapeutic efficacy of Daratumumab, as previous research had demonstrated its limited effectiveness due to NK cell fratricide [27,58,59]. To overcome this limitation, we have developed highly potent, genetically modified NAM-cultured NK cells that are resistant to the fratricide effect caused by Daratumumab.

Following the CD38 knock-out, we achieved a remarkable reduction of approximately 90% in CD38 expression. This genetic modification exhibited an exceptional safety profile, characterized by exceptionally rare off-target events. While CD38 knock-out alone eliminated fratricide, we have observed that the cells did not exhibit a sufficiently potent anti-tumoral activity in vitro. This manifested in a mild increase in the specific lysis of RPMI8266 target cells, with a mild reduction in measured activation markers. This reduction in activation marker expression may be attributed to the decrease in their fratricide-induced expression of measured activation markers. Subsequently, we explored the possibility of introducing our customized CAR into NAM-cultured NK cells to target CD38 expressed on MM cells using mRNA electroporation. Although this method induces transient expression of proteins, it is highly efficient and inflicts minimal stress on treated cells. These advantages, combined with the limited persistence of NK cells in patients [60], led to the selection of this delivery method over alternative options. The anti-CD38 CAR was introduced into NAM-cultured NK cells without any genomic manipulation.

To understand the dynamics of cell growth and mortality in the culture, considering of the possibility of fratricide, and to assess the feasibility of utilizing anti-CD38 CAR-expressing NK cells without CD38 deletion as a cell therapy agent, we designed a study composed of two consecutive specific lysis assays performed at two timepoints post-electroporation. The findings of this study underscore the indispensable nature of CD38 deletion in realizing the therapeutic potential of anti-CD38 CAR-expressing NK cells for the treatment of multiple myeloma. Through a comprehensive analysis of NK cell dynamics and interactions, our results have demonstrated that the mere expression of the anti-CD38 CAR, without CD38 deletion, renders these NK cells largely impotent for therapeutic purposes. This conclusion is firmly grounded in the vast fratricide observed upon CAR introduction. This phenomenon not only drastically reduced the viability of these NK cells but also fundamentally altered their functionality. It is noteworthy that similar works attempting to express anti-CD38 CAR on NK cells had avoided using anti-CD38 CAR-expressing NK cells as a control in their experiments, likely due to similar challenges and observations [46,47].

It was clear that both genetic manipulations are required for the establishment of a potent, fratricide-resistant NK cell. Indeed, combining both features resulted in NK cells showing great promise in vitro. One fundamental advantage gained by combining CD38 knock-out and anti-CD38 CAR expression was the complete clearance of CD38-expressing NK cells in the culture. It appears that during the cellular recuperation stage following electroporation, the emerging CAR-expressing NK cell population swiftly eliminates all remaining CD38 NK cells left unaffected by the preceding CRISPR editing of CD38. Functional assays in three different MM cell lines showed a remarkable killing capacity, degranulation, and pro-inflammatory cytokine expression, which corresponded well to CD38 expression levels over target cells. A non-MM cell line K562, which is CD38-negative, had target cell lysis levels comparable to those measured in other NK cell groups, implying that the activity mediated by natively expressed NK cell receptors was maintained. This is highly important, since one major advantage of genetically modified NK-cell-based therapies over the T-cell-based alternatives is the presence of a natively expressed repertoire of activating receptors directed to identify stress-induced and cancer-associated ligands.

The selective pressure exerted on the cancer cell population by CAR-expressing cells may result in a form of immunoediting which is manifested in the loss of targeted cancer antigens [57]. This may result in the re-establishment of a resistant cancer cell population. Due to its dependence on an exclusive CAR-mediated activation axis, such a process may render CAR T cells completely disabled [61,62]. Alternatively, CAR NK cells, expressing their innate repertoire of activating receptors, may still maintain their activity via these receptors. To evaluate the integrity of natively expressed NK receptors, we tested the expression levels of both activating and inhibiting NK-expressed receptors using flow cytometry. Additionally, we selected three receptors imperative for NK cell anti tumoral activity and tested their exclusive effect over activation markers expressed by NK cells. Both MICA- and B7H6-mediated activities, to test NKG2D and NKp30, respectively, showed comparable performance between all tested NK groups. This was in complete agreement with the results obtained in CD38-negative target cells (i.e., K562) and demonstrated that CD38 knock-out CAR NK cells maintained their overall potency in the absence of its anti-CD38 CAR target. Since K562 cells are known to express high levels of NKG2D ligands as well as B7H6 [63,64,65,66], our findings further validate that both receptors remained intact and functional.

Due to its central role in mediating ADCC, CD16 (another NK-expressed receptor) is a key factor in allowing combination therapy with mAbs targeting MM. There is a potential in combining CD38 knock-out CAR NK cells with mAb-mediated ADCC to produce a synergistic effect far greater than the singular effect of each one separately [67,68]. The possibility of combination therapy using a monoclonal antibody targeting BCMA or SLAM-F7, which already have several approved mAbs in the clinic, may prove to be highly efficient. Indeed, we singled out CD16 and tested its ability to activate NK cells over immobilized antigens complexed with human IgG1 mAbs. The results showed potent signaling mediated by CD16, comparable to other tested NK cell groups. Lastly, we have demonstrated anti-CD38 CAR activity in an immobilized antigen setting. This was demonstrated both in the presence and absence of the anti-CD38 mAb, Daratumumab. NK cells showed no activation induced by direct interaction with CD38. Remarkably, Daratumumab-mediated interaction peaked at the lowest CD38 concentration and plateaued throughout the CD38 concentration gradient. CD38 knock-out CAR NK cells, on the other hand, showed a perfectly linear trend, reaching an activation marker expression far greater than that recorded with Daratumumab.

Looking ahead, there is tremendous potential to enhance the capabilities of our genetically modified NK cell platform. One avenue of exploration involves introducing additional genetic modifications to these cells. For instance, incorporating elements that enhance homing to the bone marrow, such as CXCR4-R334X, could potentially improve the localization and targeting of MM cells within their primary niche [69]. Moreover, enhancing the persistence of these cells through the incorporation of membrane-bound IL-15 may extend their anti-tumoral activity and reinforce their effectiveness in vivo [70]. Furthermore, we can consider means of reducing immune rejection by the patient’s immune system. Strategies such as HLA-E overexpression can be explored to mitigate potential host-versus-graft responses [71]. Incorporating these genetic modifications and exploring their synergistic effects with our existing platform represent promising directions for advancing the field of cell-based therapies in MM treatment. These innovations hold the potential to further improve patient outcomes and bring us closer to realizing the full potential of genetically engineered NK-cell-based therapies in the fight against MM.

In conclusion, our study presents a novel approach for addressing the challenges associated with CD38-targeting therapies in MM. By combining CD38 knock-out and anti-CD38 CAR expression, we have created potent, fratricide-resistant NK cells with promising in vitro anti-tumoral activity. These findings pave the way for further exploration of this approach in preclinical and clinical settings. Additionally, the potential for combination therapies, especially in conjunction with mAbs targeting MM, holds significant promise and merits further investigation.

## 4. Materials and Methods

### 4.1. Cell Culture

PBMCs were obtained by apheresis from a healthy volunteer at Hadassah (HAD) and Rambam (RAM) Medical Centers. Cells were purified by CD3-negative selection using the CliniMACS system, followed by a CD56-positive selection using MACS cell separation columns. After a short recuperation of 16–48 h at 37 °C in IL2-supplemented media, the purified cells were subjected to CRISPR cas9 editing aimed to knock out CD38. CD38 knock-out cells were then cultured for 14 days in NAM-supplemented MEMα media in the presence of irradiated feeder cells (irradiation at 40 Gy, 130 KV, 5 mA) and IL-15. The culture volume was doubled on days 6-10 using complete medium. On the day of harvest, an mRNA anti-CD38 CAR was introduced into cells by electroporation. Approximately 5 h post electroporation, the cells were cryopreserved in standard CryoStor CSB freezing media (Biolife Solutions, Bothell, WA, USA) supplemented with 10% DMSO.

### 4.2. Knock-Out

For targeting CD38, the CRISPR-Cas9 system was used. CD38 sgRNA was synthesized by Integrated DNA Technologies (IDT) Coralville, IA. The 20 bp sgRNA genomic target sequence was TGTAGACTGCCAAAGTGTAT. sgRNA (260 pmol) was complexed pre-electroporation with 104 pmol of Alt-R Cas9 protein (IDT), forming an RNP complex at a 1:2.5 molar ratio (Cas9:sgRNA). Cells were supplemented with 3.85 mM Alt-R Electroporation Enhancer (IDT, Coralville, IA, USA). Electroporation of human primary NK cells was performed in a BTX Gemini electroporator. To evaluate editing efficiency, DNA from the cells was extracted after 14 days in culture. INDEL quantification was performed via the TIDE analysis platform—version 3.3.0 (available at https://tide.nki.nl/ (accessed on 15 September 2022).

### 4.3. Off-Target Analysis

To study the specificity profile of the CD38 sgRNAs, we conducted a multi-step sequencing analysis. First, off-target site nomination was conducted using the GUIDE-Seq method [43]. The procedure was performed in HEK 293-Cas9 cells generated by IDT. The stable cell line has a single copy of the Cas9 gene and since it is neomycin-resistant, it was cultured in DMEM supplemented with 10% fetal bovine serum (FBS; Gibco, Invitrogen, Grand Island, NY, USA), 1% penicillin/streptomycin (Gibco, Carlsbad, CA, USA), and 500 mg/mL G418 (Gibco). 2-partXT gRNA (10 μM) was electroporated into HEK293-Cas9 cells along with dsODNs, a 34 bp dsDNA fragment. The cell line was electroporated by a Lonza 4D nucleofector (Lonza, Basel, Switzerland). After 72 h post electroporation, gDNA was extracted from the cells by column purification. NGS library preparation, sequencing, and operation of the GUIDE-seq software (version 1.0.2) were performed as described previously [72]. The genomic locations of the guide-seq-identified off-target sites were analyzed via the University of California, Santa Cruz (UCSC), genome browser, version GRCh38/hg38. For the quantification of off-target events in CD38 knock-out CAR NK cells, rhAmpSeq was applied. Primers were designed to flank the on-target and all off-target sites, nominated by GUIDE-seq of HEK293-Cas9 cells, following editing with Alt-R 2part XT gRNA. Primers were pooled for locus-specific RNase H2-dependent multiplex assay amplification, followed by universal PCR to add adaptor ends for NGS. PCR amplicons were sequenced on an Illumina Mi Seq instrument (v.2chemistry, 150bp paired-end reads; Illumina, San Diego, CA, USA). The sequencing data were analyzed using CRISPECTOR software (version 1.0.5) as previously described [45].

### 4.4. FACS Analysis

Antibody panels (Supplemental Table S1) were prepared in stock solutions diluted according to manufacturer instructions (0.5 µL antibody/100 µL FACS buffer (PBS, 1%BSA)). Subsequently, 100 µL from the stock mix was aliquoted onto the cells (100,000 cells/well in 96U plate), followed by incubation of the plate in the dark for 10 min at room temperature. The plate was removed, centrifuged, and washed with FACS buffer, and samples were resuspended in 200 µL of Helix viability dye prepared in FACS buffer (final dilution of 1/20,000).

### 4.5. Killing and Fratricide Assays

NK cells were co-cultured with different target cells (RPMI-8266, H929, u266, and K562) at varying E:T ratios (5:1. 2.5:1, and 1.25:1); the final volume was 250 µL E:T plus 50 µL Daratumumab (E:T 5:1, 250,000 NK cells and 50,000 target cells). All target cell lines were labeled with CFSE (Invitrogen, Carlsbad, CA, USA), according to the manufacturer’s instructions, for 3 min at 37 °C, and fetal bovine serum (FBS) was added to the cells for 1 min. The cells were washed and resuspended to a concentration of 0.5 × 10^6^/mL in MEMα medium supplemented with 10% FBS, followed by plating 100 µL of target cells in a 96 U-shape bottom plate. For RPMI cells, an ADCC assay was performed using target cells in the presence of 8 µg/mL of Daratumumab (anti-CD38) that was added onto the cells. NK cells were prepared to the correct concentration as needed, and 100 µL was subsequently plated. The plates were incubated for either 2 h (K562 cell lines) or 6 h (with RPMI-8226, H929, and U266) at 37 °C. Following incubation, the cells were washed and resuspended in 200 µL of Helix viability dye diluted in FACS buffer. The final dilution of the dye was 1:25,000. The gating strategy applied for the FACS analysis is demonstrated in Figure S3. Experiments were performed with 3 independent biological triplicates.

### 4.6. Calcein-AM Release Assay

Calcein-AM was purchased from Invitrogen (Waltham, MA, USA). Target MM cells (RPMI8226 and H929) were incubated with 2 µM of Calcein-AM for 30 min at 37 °C. After washing, target MM cells were re-suspended in complete media and incubated with effector cells at the indicated effector-to-target ratio in the presence (1 µg/mL) or absence of Daratumumab for 4 h at 37 °C in 96-well plates. After centrifugation, the culture supernatants were collected, and fluorescence was measured via a SpectraMax M5e (Molecular Device, San Jose, CA, USA). Each experiment was performed in triplicate. The percentage of specific lysis was calculated using the equation: (Fluorescence (sample) − Fluorescence (spontaneous)/(Fluorescence (maximum) − Fluorescence (spontaneous)) × 100% and expressed as the mean of triplicate samples. 

### 4.7. Potency

NK cells were co-cultured with different target cells: K562 (chronic myelogenous leukemia), RPMI-8226, h929, and u299 (MM). The E:T ratio in these experiments was 1:3 (100,000 NK cells:300,000 target cells). The target cells were washed and resuspended to a concentration of 3 × 10^6^ cells/mL in MEMα medium supplemented with Gentamicin, L-glutamine, and 10% human serum. For each target cell, 100 µL per well was plated in 96-well U-shaped-bottom plates. For RPMI cells, ADCC assays were performed using target cells in the presence of 8 µg/mL of Daratumumab and incubated at RT for 20 min. Cells without Daratumumab received medium only. Following the incubation, NK cells were prepared in the same medium as above but supplemented with anti-CD107a antibody (diluted 1:100) to a concentration of 1 × 106 cells/mL, and 100 µL of NK cells was added to the appropriate wells. Positive control wells contained NK cells treated with 1 µL Ionomycin (200 ug/mL, Merck, Rahway, NJ, USA. cat# I9657-1MG) and 1 µL PMA (10 ug/mL, Merck, cat# P1585-1 MG). The plate was then incubated for 30 min at 37 °C. Following the incubation, cells were treated with 20 µL diluted Brefeldin A (BFA) (ENZO, Farmingdale, NY, USA. cat# BML-G405-0005)/GolgiStop mix (BD, Franklin Lakes, NJ, USA. cat# 554724) according to the manufacturer’s instructions, and the plate was then incubated for an additional 5 h at 37 °C. After the incubation, the plate was removed from the incubator, and samples were washed with PBS, followed by fixable viability dye staining with Zombie viability dye (Bio Legend, San Diego, CA, USA. cat# 4253), which was used at a final dilution per well of 1:2000 (0.05 µL per well) according to the manufacturer’s instructions. The plate was incubated at RT in the dark for 20 min, washed and stained with antibodies against CD56 and CD16 (0.5 µL per 100 µL FACS buffer), and incubated for an additional 10 min in the dark at 2–8 °C. Cells were then washed twice, followed by fixation and permeabilization using an Inside Stain Kit (Miltenyi, Bergisch Gladbach, Germany. cat#130-090-477) according to the manufacturer’s instructions. After permeabilization, intracellular staining was performed with antibodies against IFNγ, TNFα, and GM-CSF (0.5 µL antibody/100 µL permeabilization buffer). The plate was incubated for 15 min in the dark at RT, washed with permeabilization buffer, and resuspended in 200 μL FACS buffer. The plate was either immediately read in the Cytoflex (CytExpert Version 2.5.0.77) or kept at 2–8 °C until the machine was available for use.

### 4.8. Single Axis Assay

Effector cells were incubated for 6 h on plates pre-coated with increasing concentrations of human recombinant CD38 mixed with a nonspecific protein (BSA). Effector cells were harvested and stained for CD107a, IFNγ, TNFα, and GM-CSF and analyzed by flow cytometry. Dead cells were excluded by Zombie violet viability dye.

### 4.9. Cryopreservation Protocol

The cells were collected and washed at harvest with 0.5% HSA-based CliniMACS PBS/EDTA buffer. Cryopreservation was performed with 20–100 × 10^6^ cells, which were transferred into a centrifuge tube and spun down. The cell pellets were resuspended in 10 mL of cryopreservation solution, CRYOSTOR (CSB) (Biolife solution), and 1 mL of DMSO (dimethyl sulfoxide, BloodStor part#410302) was added to the tube. Cell suspensions were transferred into 50 mL cryopreservation bags to undergo freezing using Planer Controlled-Rate Freezer apparatus.

### 4.10. Thawing Protocol

After removing the bags from storage in liquid nitrogen, the bags were kept for 5 min at room temperature, then were immersed in a 37 °C water bath until no ice was left. A total of 40 mL of PlasmaLyte was added to the bags; the bags were mixed by inverting them several times.

## Data Availability

Data are contained within the article or are available from the authors upon reasonable request.

## Supplementary Materials

The following supporting information can be downloaded at: https://www.mdpi.com/article/10.3390/ijms242417231/s1.
