# Supplementary material for: Nicotinamide-Expanded Allogeneic Natural Killer Cells with CD38 Deletion, Expressing an Enhanced CD38 Chimeric Antigen Receptor, Target Multiple Myeloma Cells
Source: Int J Mol Sci. 2023 Dec 7;24(24):17231. doi: 10.3390/ijms242417231 (PMC10743602; doi:10.3390/ijms242417231)
Supplement: Supplementary file 1 [file ijms-24-17231-s001.zip › ijms-2619364-supplementary.pdf]

International Journal of
Molecular Sciences

MDPI

Supplementary Figures

Figure S1

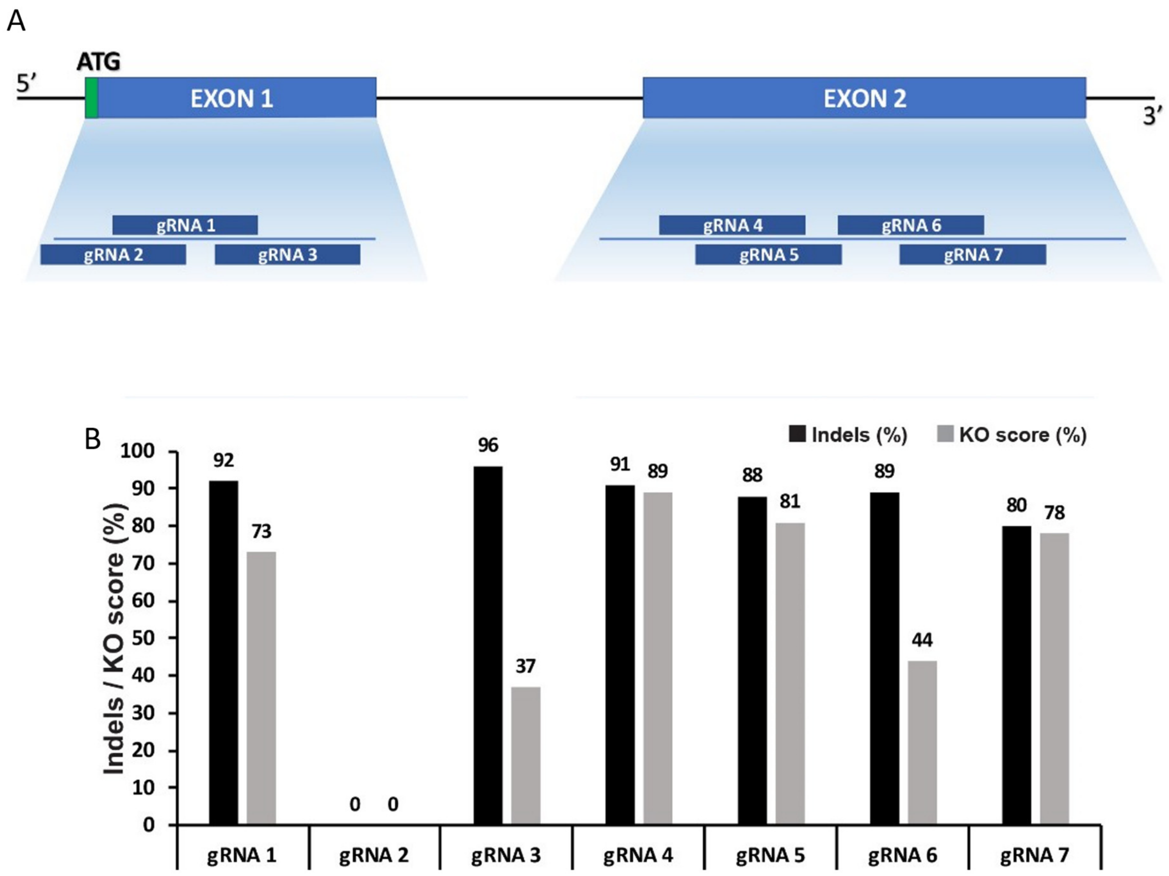

Figure S1: Experimental analysis of mutagenic NHEJ at the CD38 locus. (A) Schematic presentation of CD38 gRNAs-targets. (B) Frequency of indel mutations (black) and knock-out score (grey) in the CD38 locus determined by ICE analysis after delivery of CRISPR-Cas9 composed of 7 different gRNAs into HEK293 cells with stable expression of Cas9.

Figure S2

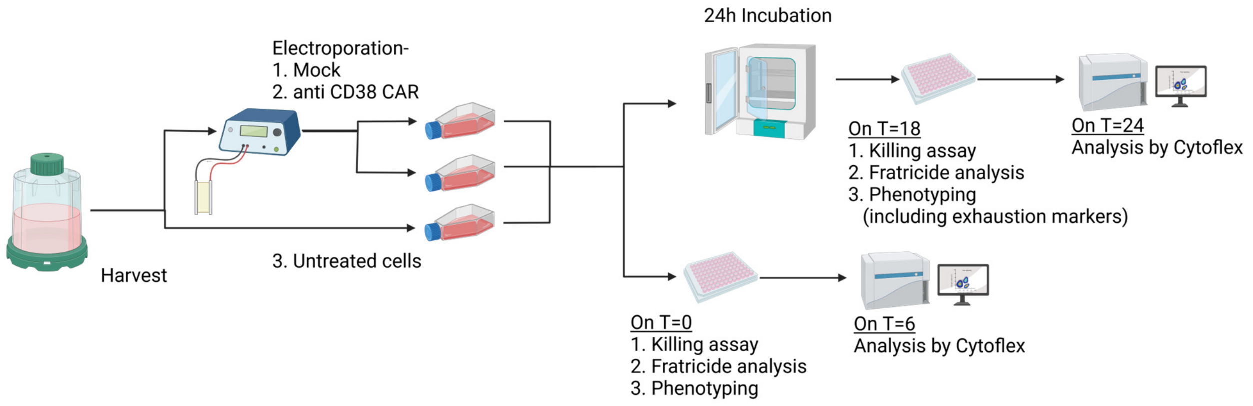

Figure S2: CD38 knock-out CAR NK cells evaluation, experimental design scheme. NK cells were cultured for two weeks in standard conditions in the presence of NAM and harvested. Cells were then either electroporated with anti-CD38 CAR mRNA, mock-electroporated, or left untreated. Cell groups were plated in 3 different flasks and incubated in a 37°C incubator for 24 hours. During incubation, cells were sampled five times and stained for viability. Lysis level in untreated cells was regarded as a control for spontaneous background lysis levels. In parallel, NK cells from each flask were sampled immediately after electroporation and 18hrs post-electroporation. Cells were subsequently cocultured with K562 (cd38-) and RPMI8266 (cd38+) for six hours in the presence or absence of Daratumumab.

Figure S3

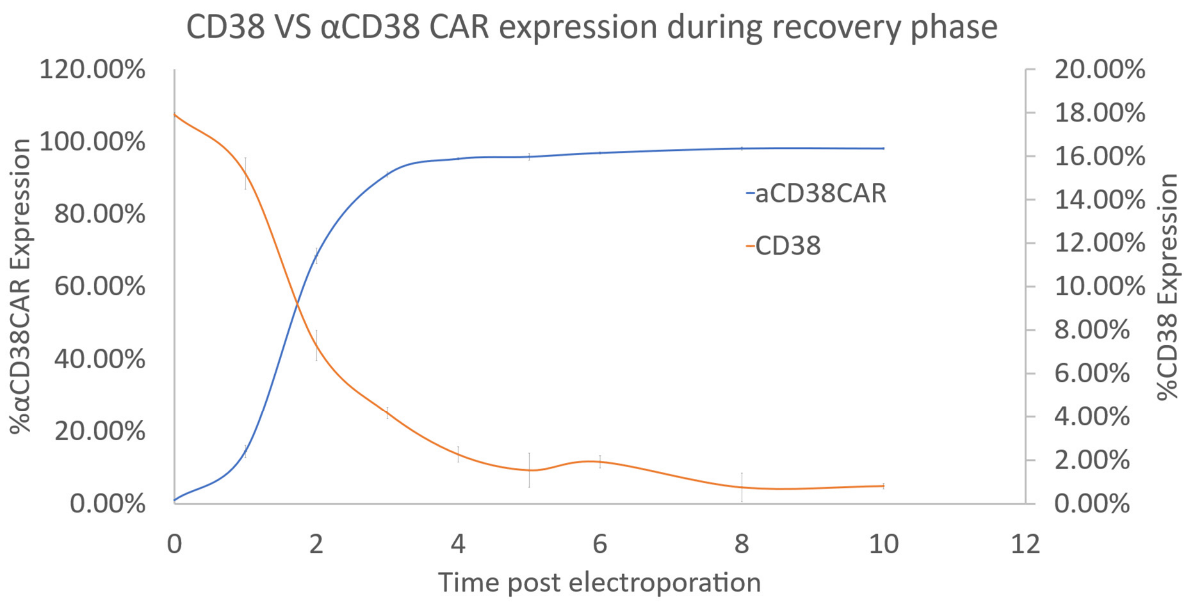

Figure S3: Longitudinal phenotype analysis of CD38 versus anti-CD38 CAR expression. On the day of harvest, 6 hours after electroporation, CD38 knock-out CAR cells were stained with FITC conjugated anti-CD56 mAb and an APC conjugated anti-CD38 mAb or incubated with His-tagged conjugated CD38 to induce complex formation with αCD38 CAR, followed by staining with FITC conjugated αHis tag mAb. Staining was followed by the addition of the Helix viability dye immediately prior to analysis by flow cytometry.

Figure S4

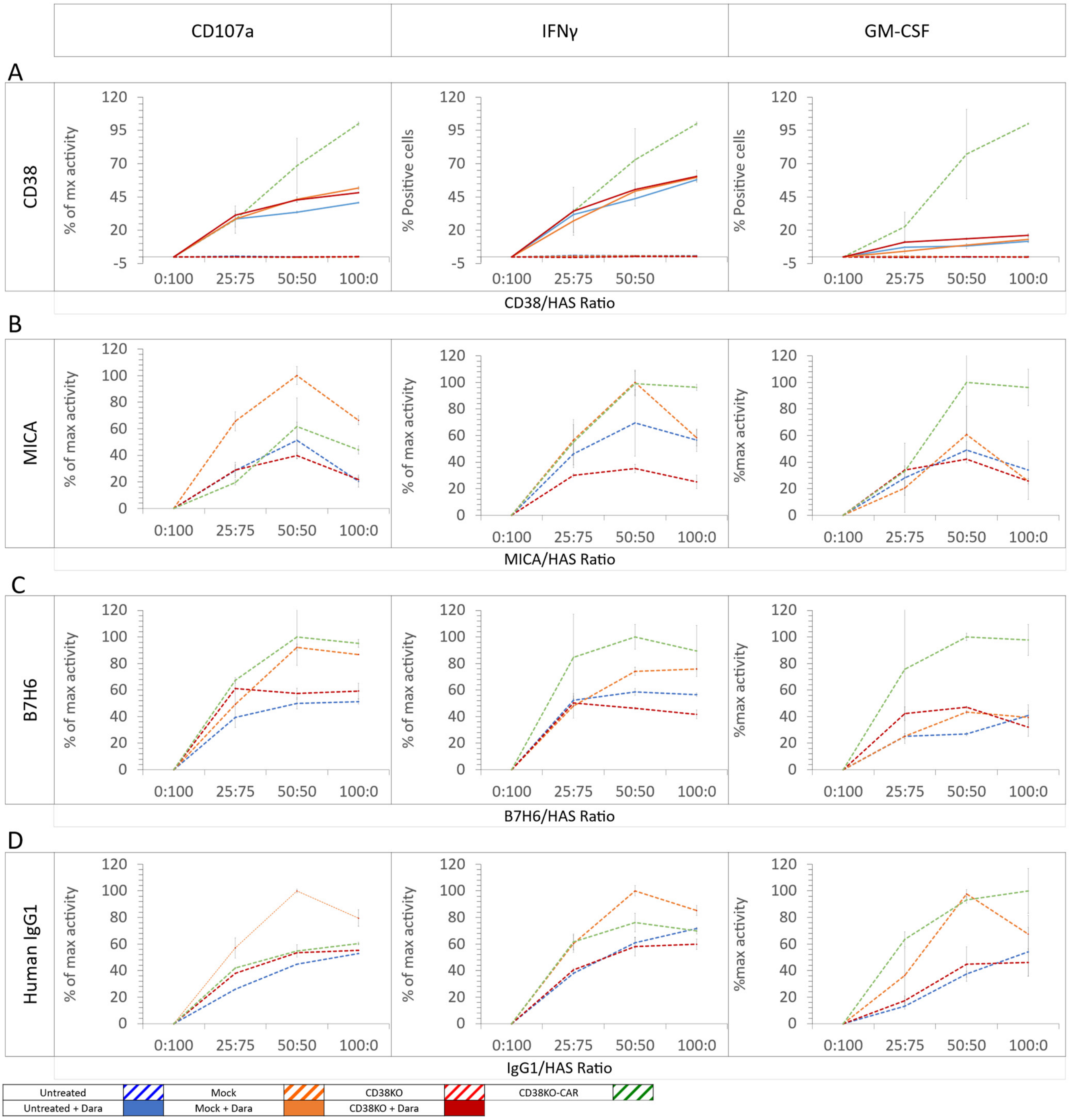

Figure S4: **Single axis activation assay.** Tissue culture plates were pre-coated with gradually increasing concentrations of **(A)** CD38 **(B)** MICA **(C)** B7H6 or **(D)** Human IgG1. CD38-coated plates were either treated with Daratumumab to induce mAb-Ag complex or left untreated. Thawed, untreated, mock electroporated, CD38 knock-out and CD38 knock-out CAR NK cells were plated on the pre-coated plates and incubated for six hours. Cells were harvested and stained for CD107a, IFNγ, TNFα, and GM-CSF and analyzed by flow cytometry. Dead cells were excluded by Zombie violet viability dye. Results shown are from 1 representative experiment of 2 performed.

Figure S5

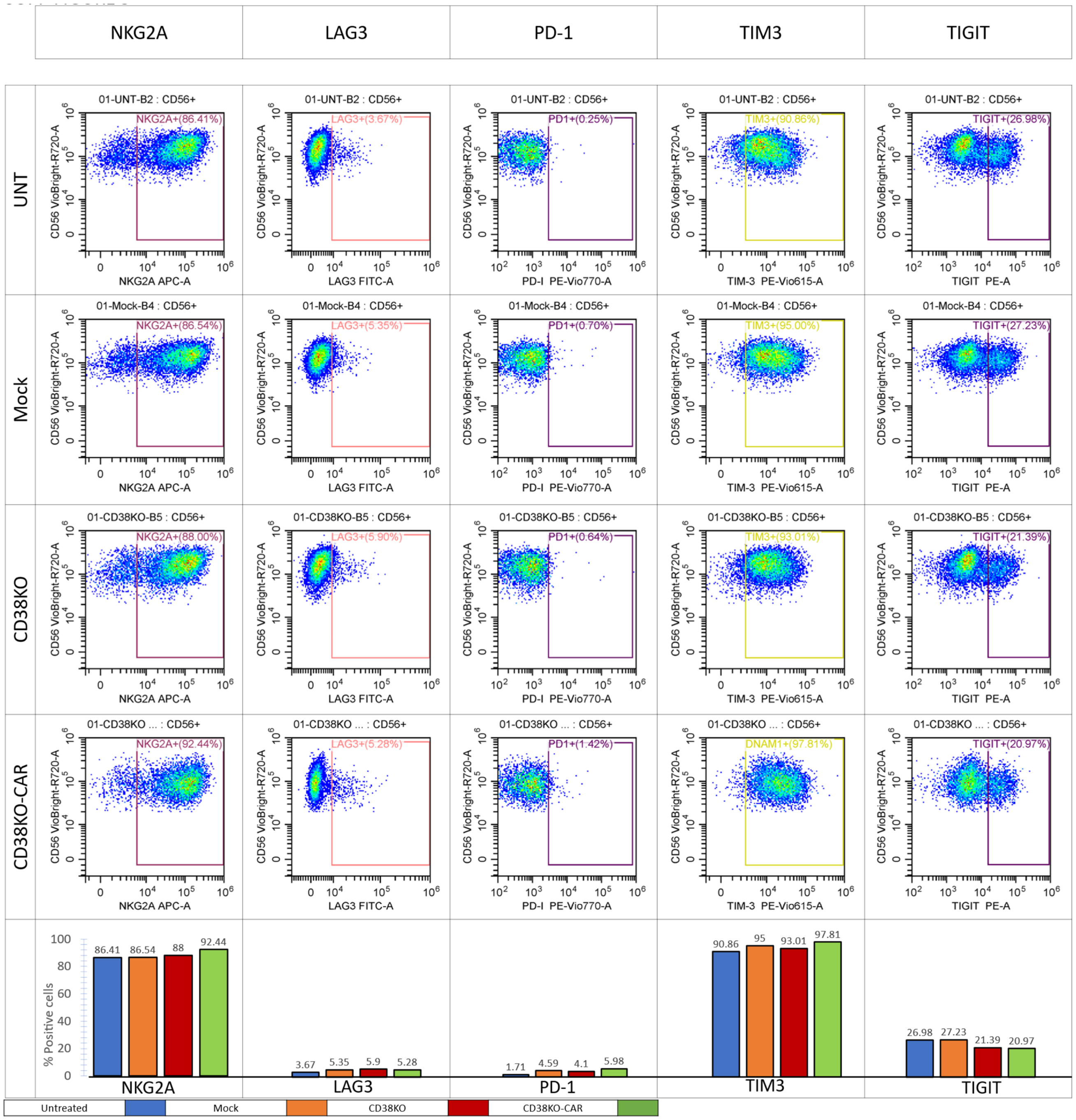

Figure S5: **Inhibition marker expression phenotyping.** On the day of harvest, 6 hours after electroporation, all effector cells were stained with a mix of fluorophore-conjugated mAbs directed to stain a panel of selected NK expressed Inhibition markers, including APC conjugated αNKG2A mAb, FITC conjugated αLAG3 mAb, PE-Vio770 conjugated αPD1 mAb, PE-Vio615 conjugated αTIM3 mAb, and PE-conjugated αTIGIT mAb. Stainings were followed by the addition of the Helix viability dye immediately prior to analysis by flow cytometry.

Table S1

	Specificity	Fluorochrome	Vendor	Cat#
Basic NK phenotyping	CD56	FITC	Miltenyi	130-114-549
	CD16	PE	Miltenyi	130-113-393
	CD3	APC	Miltenyi	130-113-135
	CD19	BV421 / VioBlue	Miltenyi	130-098-598
GDA-601 phnotyping	Anti his	FITC	abcam	Ab 1206
	CD38	APC	Miltenyi	130-113-429
	CD56	PE Vio 615	Miltenyi	130-114-550
Potency assay	Zombie Violet Viability Dye	B.V 421	Bio Legend	4253
	CD107a	VioGreen	Miltenyi	130-111-629
	CD56	PE-Vio615	Miltenyi	130-114-549
	TNF-a	PE-Vio770	Miltenyi	130-118-974
	INF-g	VioR720	Miltenyi	130-113-499
	GM-CSF	APC	Miltenyi	130-123-420
Killing assay	Viability	Helix NP Blue	Bio Legend	425305
	CellTrace CFSE	CFSE	Thermo Fisher	2266584
exhaustion markers	TIM-3	PE	Miltenyi	
	LAG-3	PE-Vio615	Miltenyi	
	CD57	APC	Miltenyi	
	PD-1	PE-VIO 770	Miltenyi	130-120-385
